# MiR-200a ameliorates peritoneal fibrosis and functional deterioration in a rat model of peritoneal dialysis

**DOI:** 10.1007/s11255-019-02122-4

**Published:** 2019-03-19

**Authors:** Xin Wei, Yi Bao, Xiaojiang Zhan, Li Zhang, Guojun Hao, Jing Zhou, Qinkai Chen

**Affiliations:** 1 Department of Nephrology, The First Affiliated Hospital of Nanchang University, Nanchang, 330006 Jiangxi China; 2 Department of Nephrology, The First Affiliated Hospital of Guizhou Medical University, Guiyang, 550001 Guizhou China; 3 Department of Nephrology, Zhongshan City People’s Hospital, Zhongshan Hospital of Sun Yat-sen University, Zhongshan, 528400 Guangdong China

**Keywords:** Peritoneal dialysis, Peritoneal fibrosis, MiR-200a, Zeb1/2

## Abstract

Peritoneal fibrosis is recognised as the main cause of the technical failure of peritoneal dialysis (PD), and currently, there are no specific and effective anti-fibrosis therapies. We have found that miR-200a is down-regulated in a rat model of PD-related peritoneal fibrosis (PF) and could inhibit transforming growth factor beta 1 (TGF-β1)-induced epithelial-mesenchymal transition (EMT) in peritoneal mesothelial cells by target ZEB1/2. However, its treatment role in vivo is still largely unclear. In this study, we examined the therapeutic potential for miR-200a on PD-related PF in a rat model of PD induced by daily infusion of 4.25% dextrose-containing dialysate. Male Sprague–Dawley rats were divided into four groups: control group, PD group, PD + miR-agomir-NC group, and PD + miR-200a-agomir group (*n* = 5 in each group). MiR-200a agomir was delivered into the peritoneum by intra-peritoneal injection on days 10 and 20 after PD. We found that treatment with miR-200a agomir significantly reduced the collagen volume fraction (CVF) of the peritoneum and prevented peritoneal dysfunction. The up-regulation of the EMT marker (decreased E-cadherin and increased α-smooth muscle actin) and extracellular matrix (fibronectin and collagen I) was significantly ameliorated by miR-200a in the PD + miR-200a-agomir group. Furthermore, we demonstrated that miR-200a inhibition of PF in vivo was associated with the suppression of ZEB1 and 2, which were proved to be the target of miR-200a in our previous study. In conclusion, results from the present study suggest that treatment with miR-200a may represent a novel and effective therapy for PD-related PF.

## Introduction

Peritoneal dialysis (PD) is a major renal replacement therapy for patients with end-stage renal disease [1, 2]. Long-term exposure to a bioincompatible dialysate containing a high glucose concentration and peritonitis, which can result in peritoneal fibrosis (PF), impairing peritoneal function and leading to system failure [3]. Furthermore, some patients develop encapsulating peritoneal sclerosis, a rare condition of excessive PF with high mortality rates [4]. To date, there has been no specific and effective therapy available to prevent or inhibit the processes of PF.

The pivotal role of epithelial-to-mesenchymal transition (EMT) in the pathogenesis of PD-related PF has been well demonstrated [5, 6]. Emerging evidence shows that high-glucose conditions of dialysate and inflammation factors elevate the expression of transforming growth factor beta 1 (TGF-β1), which is the main factor controlling fibrosis in all organs. TGF-β1 binds its receptor on the cell membrane and triggers the activation of Snail, Slug, and ZEB1-2, which play a role in repressing E-cadherin expression and inducing EMT [7–10]. However, as TGF-β1 is indispensable in maintaining homeostasis of the immune system, blocking the general effects of this pathway creates long-term problems [11, 12]. Thus, targeting its downstream-related gene(s) may represent a better approach for the treatment of PF.

MicroRNAs (miRNAs) are noncoding RNA molecules of ~ 22 nucleotides that primarily serve to inhibit gene expression at the posttranscriptional level and have important roles in a wide range of pathophysiological processes. Recent findings indicated that the miR-200 family is one of the best characterised miRNAs related to the EMT-mediated fibrosis in lung and renal diseases [13, 14]. Furthermore, in our previous study we found that miR-200a could negatively regulate TGF-β1-induced EMT by targeting ZEB1-2 in human peritoneal mesothelial cells [15]. However, the treatment role of miR-200a in vivo during PD-related PF is largely unknown. Therefore, the present study examined whether miR-200a has therapeutic potential for PF in a rat model of PD.

## Methods

### Animal models and miR-200a delivery

Male Sprague–Dawley rats (200–250 g) were purchased from the Laboratory Animal Centre of Nanchang University (NanChang, China). The rats were housed in rodent cages in a 22 °C room with a 12-h light–dark cycle with free access to standard rat chow and water in Laboratory Animal Center of Nanchang University (Nanchang, China). A rat model of PF was induced, as previously described [16]. We used random numbers table to divide rats into four groups randomly, as follows: a control group, a PD group, a PD + miR-agomir-NC group, and a PD + miR-200a-agomir group (*n* = 5 in each group). Rats in the control group received daily intraperitoneal injections of saline solution (0.9% NaCl) for 4 weeks. Rats in the PD group were injected intraperitoneally with 4.25% dextrose PD solution (Baxter, Deerfield) at 100 mL/kg daily for 4 weeks and lipopolysaccharide (LPS) at 0.1 mg/kg daily for 1 week as described in previous studies [17]. Rats in the PD + miR-200a-agomir and PD + miR-agomir-NC groups received the same daily injection as rats in the PD group and were also intraperitoneally treated with miR-200a agomir (Lot No.: 4736, GenePharm, Shanghai, China) or its negative control (agomir-NC, Lot No.: 170601, GenePharm, Shanghai, China) at a dose of 10 mg/kg at 10 and 20 days. The dose of the agomir/agomir-NC we used was referring to the previous study and the introduction manual of the agomir-miR200a/agomir-NC [18].

### Peritoneal membrane histomorphometric analysis

Paraffin sections (3 mm thick) from the anterior abdominal wall were stained with Masson’s trichrome and haematoxylin and eosin (H&E). At least five photographs at a 400x magnification were taken of each rat using a normal microscope, and image analysis was performed using the Olympus multimedia image analysis system. Collagen volume fraction (CVF%) was used to evaluate the degree of fibrosis in the peritoneum. Five views of each slice were randomly selected to analyse CVF% by two separated observers. CVF was calculated as follows: CVF = collagen area/view area × 100%.

### Real-time PCR

Total miRNA were isolated from peritoneal tissues with the mirVana miRNA isolation kit (Thermo Fisher Scientific). Expression of miRNAs was detected by the TaqMan microRNA assay (Applied Biosystems, Foster City, CA), according to manufacturer’s instructions. MiR-200a and U6 primer sets were purchased from Applied Biosystems. Levels of miRNAs were normalised to U6 snRNA in each sample, and relative expression was calculated using the 2^−∆∆CT^ method, based on the mean CT value. Three independent experiments were performed, and the results are presented as means ± SD.

### Peritoneal function

Peritoneal membrane function was evaluated by a 4-h peritoneal equilibration test, as previously described [19]. Briefly, for the peritoneal ultrafiltration rate, 25 mL of 4.25% dextrose PD solution was intraperitoneally injected into each rat before the animal was euthanised. Four hours later, the peritoneal fluid was removed for ultrafiltration measurement. Net ultrafiltration was the volume of fluid removed after 4 h minus the volume of fluid administered. For the glucose transportation assay, we used D/D0 of glucose, where D is the glucose concentration in the dialysate after 4 h, and D0 is the glucose concentration in the PD solution before instillation into the peritoneal cavity.

### Immunofluorescence

To examine the expression level of EMT markers during PF, two-colour immunofluorescence was performed on the snap-frozen peritoneal tissue sections. Sections were incubated with Cy3-conjugated antibody against α-SMA (Boster, Wuhan, China) and a rabbit anti-mouse E-cadherin (Boster, Wuhan, China) overnight at 4 °C, followed by the goat anti-rabbit fluorescein isothiocyanate (FITC)-conjugated immunoglobulin G (IgG) (Boster, Wuhan, China) diluted 1:50 for 1 h. Nuclei were counterstained with 4,6-diamino-2-phenylindole (DAPI). Images were collected and analysed with a Zeiss LSM 510 Confocal Imaging System (Zeiss, Jena, Germany).

### Western blotting

Protein from peritoneal tissues was extracted as previously reported [20, 21]. Protein expression was analysed by western blot analysis with primary antibody against collagen I (Col-I) (Boster, Wuhan, China), fibronectin (FN) (Abcam, Cambridge, UK), E-cadherin (Abcam, Cambridge, UK), α-SMA (Abcam, Cambridge, UK), zinc-finger-enhancer binding protein 1 (ZEB1) (Proteintech, Wuhan, China), ZEB2 (Abcam, Cambridge, UK), or glyceraldehyde-3-phosphate dehydrogenase (GAPDH) (Boster, Wuhan, China) and then incubated with an appropriate secondary antibody. After washing, the protein was visualised with Super Signal Western Pico chemiluminescent substrate (Pierce, Rockford, IL). The signals were detected by the LiCor/Odyssey infrared image system (LI-COR Biosciences, Lincoln, NE, USA) and quantified by Image J software (National Institutes of Health). The ratio for the protein of interest was normalised against GAPDH and expressed as mean ± standard deviation (SD).

### Statistical analysis

Data were presented as mean ± standard deviation (SD) of at least three independent experiments. Statistically significant differences among groups were analysed by one-way analysis of variance (ANOVA) or Student’s *t* test using SPSS18.0 software. The differences between PD or PD + miR-agomir-nc or PD + miR-200a-agomir group versus control group in CVF, miR-200a level, ultrafiltration, D/D0 and relative protein levels were used *t* test analysis. The differences between these four groups were test with ANOVA. A difference with a *P* < 0.05 was considered to be statistically significant.

## Results

### MiR-200a gene transfer prevents PF in a rat model of PF

We have done the post hoc analysis to determine the power of this study; the statistical power for t test and ANCOVA analysis is 0.107 and 0.114, respectively.

**Fig. 1:**
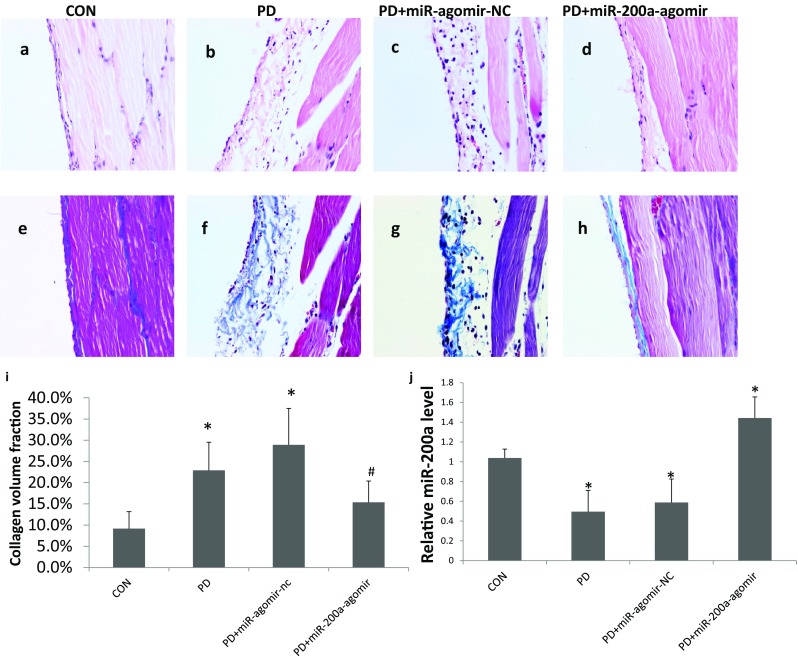
miR-200a treatment decreases PD-induced peritoneal membrane thickness and fibrosis in rats. **a**–**d** Haematoxylin–eosin (HE) staining of the peritoneal membrane. **e**–**h** Masson’s trichrome staining (Masson) of peritoneal membrane. Original magnification × 400. **i** Changes in collagen volume fraction (CVF) in each group of rats. **j** The expression of miR-200a in each group of rats. CON, normal control group; PD, PD model group; PD + miR-agomir-NC, PD model rats treated with scramble control miR; PD + miR-200a-agomir, PD model rats treated with miR-200a agomir. CVF = collagen area/view area × 100%. *P < 0.05 versus the control group, ^#^*P* < 0.05 versus the PD group. Data represent means ± SD for five rats in each group

As shown in Fig. 1, the peritoneum of the control rats was composed of a monolayer of mesothelial cells with a thin layer of connective tissue underneath. Compared with the control group, the peritoneal tissue of the PD and PD + miR-agomir-NC groups showed significant loss of the mesothelial cell monolayer and thickening of the submesothelial compact zone. In contrast, miR-200a treatment significantly reduced the peritoneal thickening (Fig. 1a–h). To assess the degree of fibrosis, we used CVF to quantify the collagen content in peritoneal tissues. Compared with the control group, CVF in the PD and PD + miR-agomir-NC groups was significantly higher (0.229 ± 0.062 vs. 0.092 ± 0.040, *P* < 0.05; 0.289 ± 0.086 vs. 0.092 ± 0.040, *P* < 0.05, respectively). However, there was no significance difference between CVF in the control group and the miR-200a treatment group. The CVF in the miR-200a treatment group was significantly decreased, when compared with the PD group (0.154 ± 0.050 vs. 0.229 ± 0.062, *P* < 0.05) (Fig. 1i).

### MiR-200a gene transfer ameliorated peritoneal transport function

**Fig. 2:**
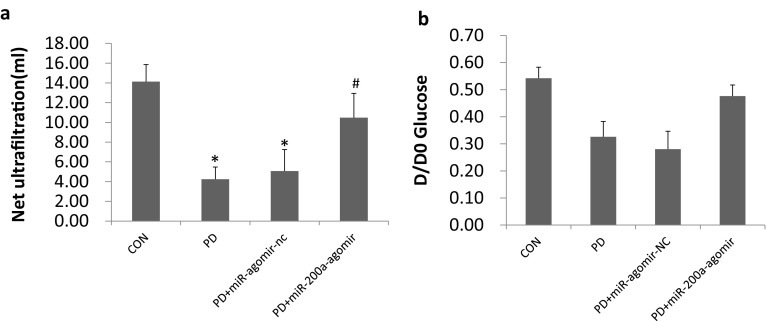
miR-200a treatment protect peritoneal function in rats model of PD. **a** The net ultrafiltration rate was tested to assess the solution transport function of peritoneum in different groups. **b** The transfer of glucose (D/D0) was assayed in different group to evaluate the solute transport function of peritoneum in different groups. CON, normal control group; PD, PD model group; PD + miR-agomir-NC, PD model rats treated with scramble control miR; PD + miR-200a-agomir, PD model rats treated with miR-200a agomir. *P < 0.05 versus the control group, ^#^*P* < 0.05 versus the PD group. Data represent means ± SD for five rats in each group

To evaluate whether the morphological preservation induced by miR-200a treatment had an impact on preserving peritoneal function, ultrafiltration (UF) and glucose D/D0 ratio (the ratio of 4-h to 0-h glucose concentration of the dialysate) were measured to assess the water transport and small solutes transport in the peritoneum. It was demonstrated that UF in the PD and PD + miR-agomir-NC groups were significantly decreased (4.235 ± 1.247 vs. 14.123 ± 1.735, *P* < 0.05; 5.072 ± 2.170, *P* < 0.05, respectively) (Fig. 2a). However, miR-200a treatment largely prevented such loss of peritoneal function, as the UF in the miR-200a treatment group was more than two times of that in the PD group (10.469 ± 2.471 vs. 4.235 ± 1.247, P < 0.05) (Fig. 2a). Further, the mass transport of glucose did not reach statistical significance between the groups. D/D0 in the miR-200a treatment group was partially recovered, as shown in Fig. 2b.

### MiR-200a gene transfer ameliorated PF by attenuating the expression of fibrotic and EMT markers

**Fig. 3:**
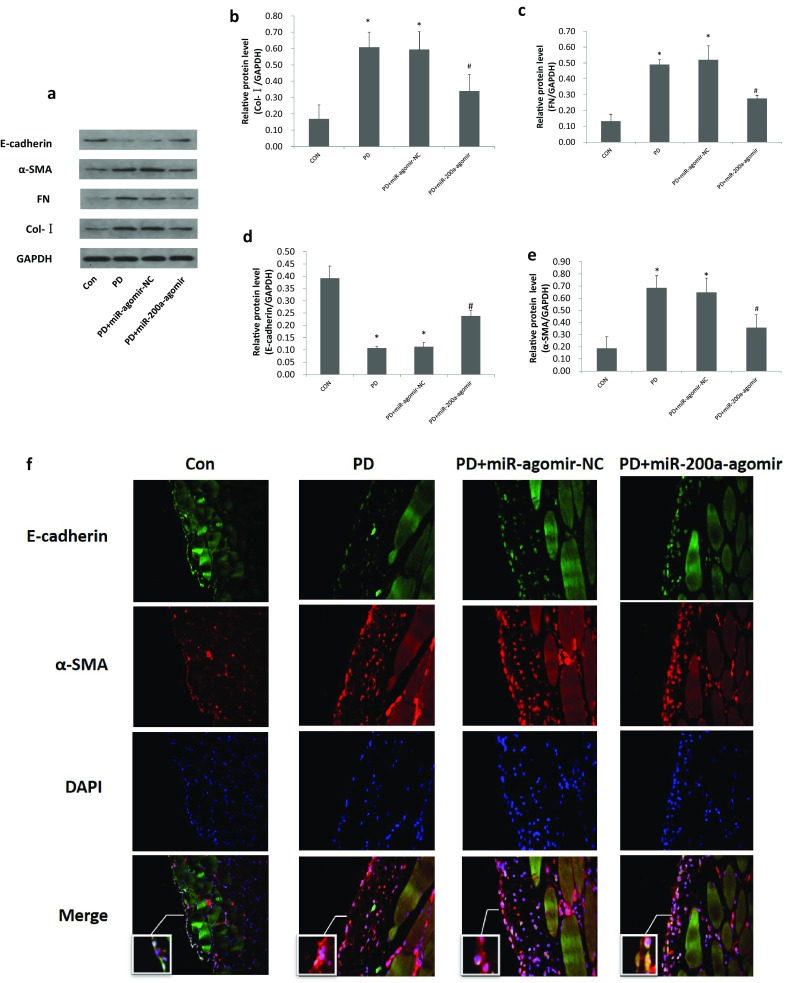
miR-200a treatment in rat PD model halts peritoneal fibrosis as demonstrated by inhibition of COL-I, FN deposition and EMT. **a** Western blot shows the expression of COL-I, FN, α-SMA and E-cadherin in peritoneum of different groups. **b**–**e** Quantitative analysis of relative protein levels of E-cadherin, α-SMA, COL-I and FN in different groups (normalised to GAPDH). F: Two-colour immunofluorescence of anterior abdominal wall sections detects that few α-SMA positive cells were shown in normal peritoneum, α-SMA positive cells (red) were increased in PD and PD + miR-agomir-NC groups, however, which were decreased in PD + miR-200a-agomir group. In contrast, the expressions of E-cadherin (green) in peritoneum were decreased in PD and PD + miR-agomir-NC groups. The changes in expression level ofα-SMA and E-cadherin in peritoneal mesothelial cells were further illustrated in the inserted small pictures. Nuclei are stained by DAPI (blue). Original magnification, × 400. Data represent means ± SD of five rats in each group. CON, normal control group; PD, PD model group; PD + miR-agomir-NC, PD model rats treated with scramble control miR; PD + miR-200a-agomir, PD model rats treated with miR-200a agomir, *P < 0.05 versus the control group, ^#^*P* < 0.05 versus the PD group

The protective effects of miR-200a on PF were further demonstrated by the ability of overexpressing miR-200a to inhibit the protein expression level of fibrotic markers such as Col-I and FN. As shown in Fig. 3a–c, the relative protein level of Col-I and FN in the peritoneum were significantly higher in the PD group than in the control group (*P* < 0.05), but miR-200a treatment reduced these levels to nearly the control levels. Furthermore, we found such protective effect was associated with the blockade of EMT by preventing the loss of the epithelial marker E-cadherin, and the gain of the mesenchymal markerα-SMA (Fig. 3a, d, e). Similar results were obtained in the immunohistochemistry analysis of the peritoneum (Fig. 3f). Compared with the control group, the expression levels of E-cadherin in the PD and PD + miR-agomir-NC groups were significantly decreased (*P* < 0.05), and the levels of α-SMA were significantly increased (*P* < 0.05), but such change disappeared in the miR-200a transfer group.

### MiR-200a protected against PF, possibly by targeting ZEB1 and ZEB2

**Fig. 4:**
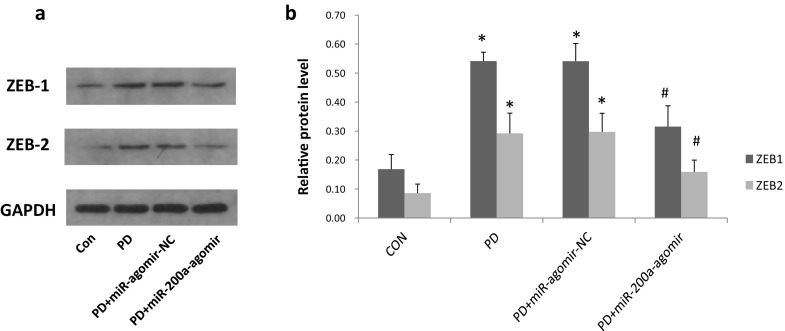
miR-200a treatment inhibits the expression of ZEB1 and ZEB2 in peritoneum of PD rats. A: Western blot shows the expression of ZEB1 and ZEB2 in peritoneum of different groups. B: Quantitative analysis of relative protein levels of ZEB1 and ZEB2 (normalised to GAPDH). Data represent means ± SD of five rats in each group. CON, normal control group; PD, PD model group; PD + miR-agomir-NC, PD model rats treated with scramble control miR; PD + miR-200a-agomir, PD model rats treated with miR-200a agomir, *P < 0.05 versus the control group, ^#^*P* < 0.05 versus the PD group

We have previously proved that miR-200a could suppress the EMT by targeting ZEB1 and ZEB2 in vitro [15]. In this study, we investigated whether miR-200a transfer could inhibit the expression of ZEB1 and ZEB2 in vivo. As expected, we found the expression level of ZEB1 and ZEB2 were significantly increased in the PD and PD + miR-agomir-NC groups when compared with the control group (*P* < 0.05). However, these levels were markedly lower in the PD + miR-200a-agomir group than those in the PD and PD + miR-agomir-NC groups (*P* < 0.05) and showed no difference between the PD + miR-200a group and control group (Fig. 4).

## Discussion

PD as an alternative renal replacement therapy for patients with end-stage renal failure, and it has been developing rapidly worldwide [22]. However, long-term exposure to PD fluid causes a progressive PF characterised by thickness of the peritoneum, increased extracellular matrix deposition, and loss of mesothelial cells [23]. Therefore, it is important to develop therapeutic strategies for PF. Accumulating evidence has shown that microRNA plays a key role in inhibiting the progression of PD-related PF [17, 19, 24].

In our previous study, we found that miR-200a was down-regulated in the PD rat model with PF. In vitro, TGF-β1-induced EMT was also associated with down-regulation of miR-200a but up-regulation of ZEB1/2 in human peritoneal mesothelial cells. Finally, we demonstrated that miR-200a could inhibit TGF-β1-induced EMT by targeting ZEB1/2, which are transcriptional repressors of E-cadherin [15]. In the present study, we used agomir which was a class of chemically engineered oligonucleotides act as mimic of microRNA to overexpress the level of miR-200a in rats model of PD, we proved that agomir-miR-200a was capable of intervening in progressive PF and protecting of functional injury. Though the changes in D/D0 have not shown the statistical significance, it may be associated with individual variation in the glucose metabolic rate and solute transport efficiency, which was consistent with previous studies in human and animal [25]. Furthermore, it was shown that up-regulated miR-200a was associated with down-regulation of ZEB1/2 and inhibition of EMT, which was consistent with our previous study in vitro. Results from this study suggested that miR-200a plays a protective role and has therapeutic potential for PD-related PF.

Consistent with the previous finding that miR-200a is a key player in a variety of disease models associated with fibrosis, including pancreatic fibrosis [26], liver cirrhosis [27], and obstructive nephropathy [14], Xu et al. found that miR-200a was down-regulated in TGF-β1-activated pancreatic stellate cells and miR-200a mimic reversed the increased expression of mesenchymal markers vimentin and ECM proteins by activating the PTEN/Akt/mTOR pathway [26]. In the process of liver fibrosis, Yang et al. found that overexpression of miR-200a reduced the SIRT1 expression, consequently preventing activation and proliferation of hepatic stellate cells [28]. Xiong et al. demonstrated that miR-200a and miR-141, two members of the miR-200 family, were down-regulated at the early phase of unilateral ureteral obstruction (UUO). TGF-β1 induced tubular EMT in vitro, and it was also found that miR-200 family was responsible for protecting tubular epithelial cells from mesenchymal transition by target suppression of ZEB1/2. ZEB1/2 are recognised as crucial genes during the regulation of EMT. We have found that ZEB1/2 were the direct targets of miR-200a during the process of EMT in our previous study in vitro. In the present study, we also found that restored peritoneal miR-200a suppresses the expression of ZEB1/2 in rat peritoneum, confirming the regulation relationship between miR-200 and ZEB1/2. Our study has several limitations as follow. First, we have not to investigate the source of a-SMA positive myofibroblasts; thus, there is not clear that what kind of cells, such as mesothelial cells, bone-marrow-derived cells and endothelial cells, is the target cells of miR-200a in vivo. Second, we have not used antagomir of miR-200a to silence endogenous miRNA-200a to investigate the opposite effects. Third, as an animal study, the sample size we used was limited; thus, the statistical power of this study was insufficient. In order to further confirm the function of miR-200a in peritoneal fibrosis.

In conclusion, the present study identifies that after long-term exposure to PD dialysate, PF occurs with a loss of epithelial markers. For the first time, demonstrated overexpression of miR-200a is capable of inhibiting PD-related PF and improving peritoneal dysfunction. ZEB1/2 may be the main targets by which overexpression of miR-200a inhibit PF. Results from this study suggest that treatment with miR-200a may represent a novel and effective therapy for PD-related PF.
